# Design and Analyze a New Measuring Lift Device for Fin Stabilizers Using Stiffness Matrix of Euler-Bernoulli Beam

**DOI:** 10.1371/journal.pone.0168972

**Published:** 2017-01-03

**Authors:** Lihua Liang, Mingxiao Sun, Hongyu Shi, Tiantian Luan

**Affiliations:** College of Automation, Harbin Engineering University, Harbin, Nangang District 145, Harbin city, Heilongjiang Province, China; Beihang University, CHINA

## Abstract

Fin-angle feedback control is usually used in conventional fin stabilizers, and its actual anti-rolling effect is difficult to reach theoretical design requirements. Primarily, lift of control torque is a theoretical value calculated by static hydrodynamic characteristics of fin. However, hydrodynamic characteristics of fin are dynamic while fin is moving in waves. As a result, there is a large deviation between actual value and theoretical value of lift. Firstly, the reasons of deviation are analyzed theoretically, which could avoid a variety of interference factors and complex theoretical derivations. Secondly, a new device is designed for direct measurement of actual lift, which is composed of fin-shaft combined mechanism and sensors. This new device can make fin-shaft not only be the basic function of rotating fin, but also detect actual lift. Through analysis using stiffness matrix of Euler-Bernoulli beam, displacement of shaft-core end is measured instead of lift which is difficult to measure. Then quantitative relationship between lift and displacement is defined. Three main factors are analyzed with quantitative relationship. What is more, two installation modes of sensors and a removable shaft-end cover are proposed according to hydrodynamic characteristics of fin. Thus the new device contributes to maintenance and measurement. Lastly, the effectiveness and accuracy of device are verified by contrasting calculation and simulation on the basis of actual design parameters. And the new measuring lift method can be proved to be effective through experiments. The new device is achieved from conventional fin stabilizers. Accordingly, the reliability of original equipment is inherited. The alteration of fin stabilizers is minor, which is suitable for engineering application. In addition, the flexural properties of fin-shaft are digitized with analysis of stiffness matrix. This method provides theoretical support for engineering application by carrying out finite element analysis with computers.

## 1 Introduction

The undesirable motion of ships at sea is induced by the action of environmental disturbances: waves, wind and current. Roll is particularly severe, which can not only affect safety of ships and equipments, but also greatly reduce comfort of passengers [1, 2]. Therefore, a major concern is to continue to improve roll stability in ship motion control.

During the past decades, anti-rolling technology has greatly been developed in a variety of directions. Fin stabilizers are the most widely used in active anti-rolling nowadays. The anti-rolling effect of more than 90% is obtained in theory, but it is difficult to achieve in actual project [3]. The main reason is that fin-angle feedback control is generally used in research of fin stabilizers. Since the simple and reliable measuring device located in the interior of hull is convenient for maintenance and replacement [4, 5]. However, static lift in fin-angle feedback control is calculated with fin-angle based on ideal constant hydrodynamic. Actually, there is a huge difference between dynamic and static hydrodynamic of fin stabilizers [6]. In addition, the relationship is nonlinear and uncertain between fin-angle and actual lift due to interaction of fins, bilge keels and hull induces [7, 8]. Many disturbance factors induce a large deviation between theoretical and practical value of lift. Hence, the problem in measuring actual lift is exigent to be solved.

Nowadays, lift-feedback control of fin stabilizers is still an emerging research. While there is few published literature and engineering data. Measurement of actual lift is difficult owing to atrocious ocean environment and numerous disturbance factors [9]. The stress state of fin stabilizers is complex, and installation and maintenance of sensors are redesigned in practical engineering [10]. The key of measurement is accuracy, thus determination of measurement method is a difficult point of lift-feedback control.

The most direct way of measuring lift is to install massive force sensors on fin surface. Lift is obtained by superposition of infinitesimal [11]. But sensors are easily damaged in waves and difficult to maintain. Hence, specialized measuring device is designed according to specific characteristics of fin stabilizers. Firstly, American Sperry Marine [12] first proposed the concept of lift-feedback control, and measuring lift device is installed in hollow shaft. The application has not been extended due to difficult installation and maintenance in narrow shaft [13]. Secondly, British Rolls Royce uses strain gauge in cross shaft of fin stabilizers, and loading force of strain gauge is converted to lift [14]. The shortcoming is its special position and the vulnerability of strain gauge. Maintenance is costly, since ship must be docking in demand of maintenance. The method is not universal, which is only suitable for retractable fin stabilizers. Thirdly, Japanese MITSUBISHI [15] proposes to install pressure sensors in hydraulic servo system of driving fin rotation. Lift is measured with hydrodynamic pressure-center of fin. The changes are few in this way and maintenance of device is easy. However, the required parameters are nonlinear, which are related to fin type, angle, angular velocity and ship speed [16, 17]. So the further study is needed. Finally, Research Institute of ship anti-rolling and control proposes a measuring lift method using bearing load [18, 19]. The micro install and pressure sensors are installed in the box of fin stabilizers [20–22]. While processing and assembling precisions are very high and special sensors are needed be designed [23–26]. Thus implementation was not easy to achieve in practice. A new device is designed for measuring lift, which is improved based on actual fin stabilizers and method of Sperry Marine [27–29].

The structure is composed of 6 sections in the paper. Section 2 analyses deviation induced by fin-angle feedback control in order to avoid interference factors. The new measuring device is designed and analyzed theoretically using stiffness matrix in Section 3. Section 4 introduces two installation modes of sensors. Then theoretical calculation and simulation are carried out to analyze main factors. Section 5 is results and discussion, which presents comparison of measuring lift results of fin in water tank and new fin-shaft device, and the experimental platform of lift-feedback fin stabilizer. Finally, the conclusion is given.

## 2 Analysis of deviation

### 2.1 Roll motion model

The roll motion model of ship equipped with fin stabilizers can be expressed as follow according to Conolly theory:

$$(I_{x}+\Delta I_{x})\frac{d^{2}\varphi }{dt^{2}}+B_{1}\frac{d\varphi }{dt}+B_{2}\left|\frac{d\varphi }{dt}\right|\frac{d\varphi }{dt}+C_{1}\varphi +C_{3}\varphi^{3}+C_{5}\varphi^{5}=-K_{\omega }-K_{\text{c}}$$ (1)

Where *I*_*x*_ denotes inertia of roll moment. Δ*I*_*x*_ denotes added damping inertia. *φ* denotes roll angle of ship. *B*_1_ and *B*_2_ denote damping torque coefficients. *C*_1_, *C*_3_ and *C*_5_ denote restoring torque coefficients. $B_{1}\frac{d\varphi }{dt}+B_{2}\left|\frac{d\varphi }{dt}\right|\frac{d\varphi }{dt}$ denotes damping torque of ship. *C*_1_*φ* + *C*_3_*φ*^3^ + *C*_5_*φ*^5^ denotes restoring torque of ship. *K*_*ω*_ denotes disturbance torque of waves. *K*_*c*_ denotes control torque of fin stabilizers.

**Fig 1:**
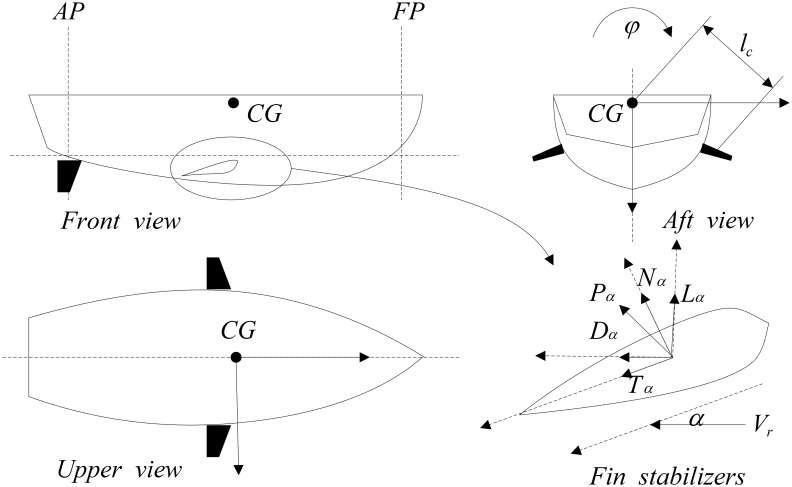
Ship and fin stabilizers.

The ship is still while disturbance torque is completely compensated by control torque. Hence, the key factor of roll reduction is the accuracy of control torque induced by fin stabilizers. Control torque is determined by disturbance torque. But the motions of waves are random and marine environment is very complex. Consequently, measuring actual disturbance torque accurately and effectively is a difficult problem. The Ship and fin stabilizers are shown in Fig 1.

The hydrodynamic force *P*_*α*_ is resolved according to structure of fin, when angle of attack is *α*. Relationships of lift *L*_*α*_, resistance *D*_*α*_, normal force *N*_*α*_ and tangential force *N*_*α*_ are shown as follow.

$$P\alpha =N\alpha^{2}+T\alpha^{2}=L\alpha^{2}+D\alpha^{2}={\left(N\alpha \text{cos}\alpha -T\alpha \text{sin}\alpha \right)}^{2}+D\alpha^{2}$$ (2)

Because lift really maintains ship stability and fins are symmetrically fixed on both sides of bilge. Then control torque can be expressed as:

$$K_{\text{c}}=2L\alpha l_{c}\text{cos}\varepsilon \approx 2L\alpha l_{c}$$ (3)

Where *l*_*c*_ is the distance between hydrodynamic pressure-center and pivot point of fin-shaft. *ε* is the included angle between fin center-line and vertical axis, which is very small and negligible.

As a result, the difficult point of fin stabilizers is how to measure actual lift accurately.

### 2.2 Analysis of reasons

Lift is constant in conditions of constant speed and fixed fin angle *α*.

$$L\alpha =\frac{1}{2}\rho V_{r}^{2}A_{F}C_{L}^{}(\alpha )$$ (4)

Where *ρ* denotes fluid density. *A*_*F*_ denotes projection area of fin. *C*_*L*_(*α*) denotes lift coefficient.

The reasons of deviation are analyzed on the basis of parameters affecting lift in Eq (4).

**Fig 2:**
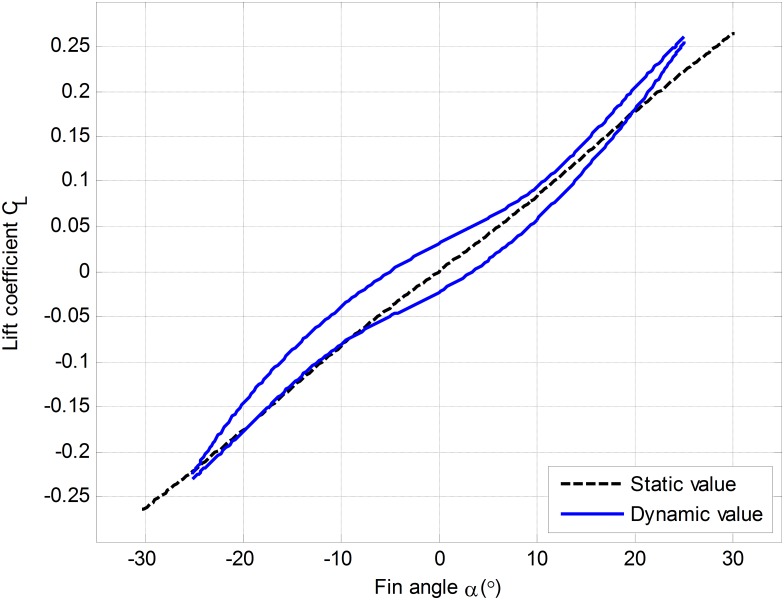
Hydrodynamic characteristics of fin stabilizers.

1. Lift coefficient *C*_*L*_(*α*). The relationship between lift and fin angle is not linear according to dynamic hydrodynamic characteristics of fin stabilizers obtained from water tank experiments. Relation curve is spindle closed, as shown in Fig 2.
  Lift characteristics become very complicated with the increase of non-dimensional frequency *Kt*, when fin is reciprocation. This leads that the edge effect is generated, while attack angle and velocity of flow on the suction surface of fin increase. So there are increased lift and delayed stall angle. Thus, the static and dynamic lift curves of fin are not consistent.
2. Flow velocity *V*_*r*_. *V*_*r*_ is a relative velocity of fluid and ship for fin moving along with ship. The measurement of relative velocity is difficult in practice. Hence, *V*_*r*_ is generally replaced by ship speed.
3. Fluid density *ρ*. *ρ* varies in different sea areas and it is changing dynamically while the ship is sailing. *ρ* is considered as a constant in theory. Moreover, dynamic hydrodynamic characteristics of fin stabilizers are disturbed by other factors. The ignored problems of fin-angle feedback control can be summarized as follow.
4. The fin angle *α*, Reynolds number *Re* and Froude number *Fr* are not constant, which are uncertain and variable with ship movement. Lift coefficient is a static coefficient obtained through water tank experiments in engineering design for fin stabilizers. Theoretical lift is difficult to meet dynamic similarity principle. As a result, the optimization is hard to achieve in design of control system.
5. The fin motion is a kind of coupled motion with forward motion, rotation and ship motion. These motions induce complex unsteady vibration, which brings great change of hydrodynamic characteristics. But the effect of factors is ignored in static estimation.
6. The influence on lift induced by roll, pitch, yaw and heave of ships can be equivalent to oblique flow angle Δ*α*. The average variance of numerous Δ*α* is calculated for irregular waves and its measurement is hard to obtain in real time at sea. The fin angle is measured in fin-angle feedback control system with Δ*α* ignored.
7. The hull and bilge keel can induce interference on hydrodynamic characteristic of fin. The flow velocity of this part in the hull boundary layer is less than flow velocity. Hydrodynamic force of fin will increase in this state, while these factors are not reflected in conventional fin stabilizers.
8. The front and rear fins may interfere with each other in the multiple fin stabilizers system. Actual hydrodynamic force will decrease, since rear fin is affected by flow of front fin.

In summary, actual lift of fin is more complex than the result of water tank experiments in Fig 1. As a result, there is a large deviation between theoretical and actual dynamic lift. The deviation of lift leads to the deviation of control torque, which is difficult to compensate disturbance torque. Thus, the roll reduction performance is reduced. In addition, the deviation not only results in unreasonable distribution of energy for system, but also induces unnecessary consumption.

## 3 Design of new measuring device

The essential defect of fin-angle feedback control causes the bottleneck for anti-rolling performance of conventional fin stabilizers. So the study of fin stabilizers should consider dynamic hydrodynamic characteristics. A simple and reliable measuring method should be found for the view of practical engineering. Lift is measured by improved fin-shaft mechanism of fin stabilizers as follows.

### 3.1 Structure design of new device

The fin-shaft is installed on the box of fin stabilizers with upper supporting of angular contact ball and lower supporting of spherical roller bearing. The box is welded on hull bilge. One end of rocker arm and fin-shaft are fixed together, and the other end is connected with hydraulic servo device. Rocker arm drives fin-shaft to rotate as the setting control mode. The fin is fixed on the outer end of fin-shaft, which stretches into sea.

**Fig 3:**
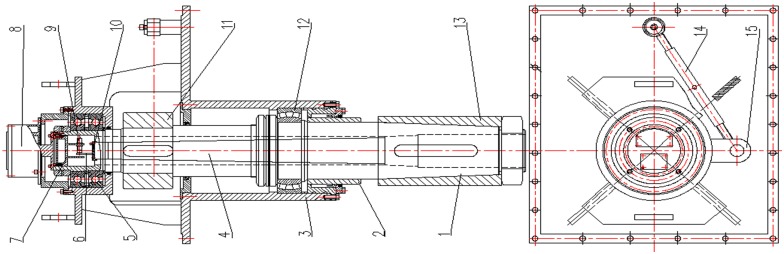
Fin-shaft combined structure of fin stabilizers.

The designed of fin-shaft is hollow, in which solid shaft-core is installed. The shaft-shell and outer end of shaft-core are fixed together, which are closely matched and rotate together. A removable shaft-end cover is on inner side of fin-shaft such as a bottle cap. Moreover, two non-contact displacement sensors are installed on the inner side of shaft-end cover along normal and tangential direction of fin. The angle sensor is installed on fin-shaft for measuring fin angle. The installment and maintenance of shaft-end cover are easy because it can be located inside of hull and be disassembled. The concrete structure is shown in Fig 3.

### 3.2 Stress analysis of new device

**Fig 4:**
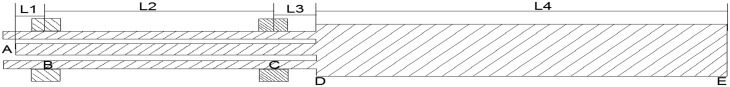
Simplified structure of fin-shaft.

The structure of fin-shaft can be divided into external shaft-shell and inner shaft-core. Shaft-shell is relatively thinner in the inner of fin-sleeve, and the other part is thicker. Shaft-core is suspended in the inner of fin-sleeve, and the other part is fixed on shaft-shell tightly, then they can be considered as a whole. The bearings form two supporting points, so *BC* of shaft-shell can be approximated as a simple beam. *CE* can be considered as a cantilever beam, since the impending shaft-shell located in the lower bearing is longer. Shaft-core is fixed at *D*, thus *AD* is a cantilever beam in the interior. The overall structure of fin-shaft is approximated as a combination of simple beam and cantilever beam. Finally, the simplified structure is shown in Fig 4.

The hydrodynamic force can be approximated as joint force acting on pressure-center of fin, when fin is rotating in waves. Fin-shaft induces tiny bending deformation under the action of hydrodynamic force, and the deformation of *E* can cause deformation of *D*. Then the deformation of *D* can make the upwarp of shaft-core in shaft-shell. The movement of shaft-core isn't bound by shaft-shell, because the space inside of shaft-shell is large enough. *AD* of shaft-core is such as the lever shaft. The slope rotation angle of *D* can be transformed to the displacement *h*_*A*_ of *A*. The section inertia *I*_*D*_ of *D* is relatively small, because section of *D* is annular. Therefore, the displacement *h*_*A*_ is relatively large, which can reduce the dependence on precision of sensors.

### 3.3 Theoretical analysis using stiffness matrix for new device

The cross section of fin-shaft is perpendicular to longitudinal axis before bend according to Euler-Bernoulli beam theory. The fin-shaft is still plane and perpendicular to longitudinal axis in condition of small bend. This case can only happen when fin-shaft is under simple couple stress or torque in theory, but the assumption is reasonable in practical engineering application. In addition, the equations are very accurate in predicting flexural properties based on Euler-Bernoulli beam theory. This theory has been verified in mechanical equipments, architecture, bridge and many other engineering fields.

The simple equation of deflection curve can not be applied directly due to complex structure of fin stabilizers. The complicated differential equations derivation can be avoided using stiffness matrix to analyze flexural properties of fin-shaft. Meanwhile, deflection and slope rotation angle of fin-shaft can be digitized, which can be convenient for finite element mathematical modeling and verification using computers. The analysis of fin-shaft using stiffness matrix provides theoretical support for later engineering design, modify and so on.

**Step 1 Selection of element type**
  *L* is set length of fin-shaft, and nodes are numbered by elements. Two points are assumed, which deflections, slope rotation angles and stress forces are respectively expressed as *ϕ*_*i*_, *m*_*i*_, *f*_*iy*_.
**Step 2 Selection of displacement function**
  The transverse displacement along element length is assumed as:
  $$v(x)=a_{1}x^{3}+a_{2}x^{2}+a_{3}x+a_{4}$$ (5)
  The cubic displacement function is appropriate with four degrees of freedom, because each node has a transverse displacement *v*_*i*_ and a small slope rotation angle *ϕ*_*i*_. Besides, the cubic displacement function can meet principles Euler-Bernoulli beam bending theory, continuous conditions of displacements and slope rotation angles at the joints.
  Eq (5) is expressed as functions of node freedom degrees:
  $$\begin{array}{l} v(0)=v_{1}=a_{4}\\ \frac{dv(0)}{dx}=\phi_{1}=a_{3}\\ v(L)=v_{2}=a_{1}L^{3}+a_{2}L^{2}+a_{3}L+a_{4}\\ \frac{dv(L)}{dx}=\phi_{2}=3a_{1}L^{2}+2a_{2}L+a_{3} \end{array}$$ (6)
  Then:
  $$v=[\frac{2}{L^{3}}(v_{1}-v_{2})+\frac{1}{L^{2}}(\phi_{1}-\phi_{2})]x^{3}+[-\frac{3}{L^{2}}(v_{1}-v_{2})-\frac{1}{L}(2\phi_{1}-\phi_{2})]x^{2}+\phi_{1}x+v_{1}$$ (7)
  Eq (7) is expressed as in matrix form:
  $$v=[N][d]=[\begin{array}{cccc} N_{1} & N_{2} & N_{3} & N_{4} \end{array}]{\left[\begin{array}{cccc} v_{1} & \phi_{1} & v_{2} & \phi_{2} \end{array}\right]}^{{}^{T}}$$ (8)
  Where $N_{1}=\frac{1}{L^{3}}(2x^{3}-3x^{2}L+L^{3})$, $N_{2}=\frac{1}{L^{3}}(x^{3}L-2x^{2}L^{2}+xL^{3})$, $N_{3}=\frac{1}{L^{3}}(-2x^{3}L+3x^{2}L)$, $N_{4}=\frac{1}{L^{3}}(x^{3}L-x^{2}L^{2})$.
  *N*_*i*_ is shape function, which is cubic-Hermite interpolation function. *N*_1_ = 1, while calculating Node 1. *N*_1_ = 0, while calculating Node 2. *dN*_2_ / *dx* = 1 can be derived from Eq (8) at the calculation of Node 1, because *N*_1_ is related to *ϕ*_1_. The shape functions *N*_3_ and *N*_4_ have similar effects on Node 2.
**Step 3 Definition of relationship between strain and stress**
  The cross section is flat before fin-shaft is deformed, which is still flat with a small slope rotation angle after deformed according to Euler-Bernoulli beam theory. Eq (9) can be obtained:
  $$\begin{array}{l} \varepsilon_{x}(x,y)=\frac{du}{dx}\\ u=-y\frac{dv}{dx}\\ \varepsilon_{x}(x,y)=-y\frac{d^{2}v}{dx^{2}} \end{array}$$ (9)
  The formula of bending stress can be obtained by Hooke law *σ*_*x*_ = *Eε*_*x*_ and Eq (9).
  $$\sigma_{x}=-\frac{My}{I}$$ (10)
**Step 4 Stiffness matrix equations of element**
  The bending moment and shear force are related with lateral displacement function. There are the following relations:
  $$\begin{array}{l} m(x)=EI\frac{d^{2}v}{dx^{2}}\\ V=EI\frac{d^{3}v}{dx^{3}} \end{array}$$ (11)
  Where *V* denotes concentrated loaded. *E* denotes modulus of elasticity. *I* denotes section inertia.

Joints, shear forces and bending moments are joined:

$$\begin{array}{l} f_{1y}=V=EI\frac{d^{3}v(0)}{dx^{3}}=\frac{EI}{L^{3}}(12v_{1}+6L\phi_{1}-12v_{2}+6L\phi_{2})\\ m_{1}=-m=-EI\frac{d^{2}v(0)}{dx^{2}}=\frac{EI}{L^{3}}(6Lv_{1}+4L^{2}\phi_{1}-6v_{2}+2L^{2}\phi_{2})\\ f_{2y}=-V=-EI\frac{d^{3}v(0)}{dx^{3}}=\frac{EI}{L^{3}}(-12v_{1}-6L\phi_{1}+12v_{2}-6L\phi_{2})\\ m_{2}=m=EI\frac{d^{2}v(0)}{dx^{2}}=\frac{EI}{L^{3}}(6Lv_{1}+2L^{2}\phi_{1}-6Lv_{2}+4L^{2}\phi_{2}) \end{array}$$ (12)

Eq (12) is expressed in matrix form Eq (13).

$$\left[\begin{array}{c} f_{1y}\\ m_{1}\\ f_{2y}\\ m_{2} \end{array}\right]=\frac{EI}{L^{3}}\left[\begin{array}{cccc} 12 & 6L & -12 & 6L\\ 6L & 4L^{2} & -6L & 2L^{2}\\ -12 & -6L & 12 & -6L\\ 6L & 2L^{2} & -6L & 4L^{2} \end{array}\right]\left[\begin{array}{c} v_{1}\\ \phi_{1}\\ v_{2}\\ \phi_{2} \end{array}\right]=\frac{EI}{L^{3}}\left[K\right]\left[\begin{array}{c} v_{1}\\ \phi_{1}\\ v_{2}\\ \phi_{2} \end{array}\right]$$ (13)

The relationship of force, bending moment, deflection and slope rotation angle is established through stiffness matrix [*K*]. The axial effect is ignored since the length *L* and height *h*_*L*_ of fin-shaft are relatively large. The order of deflection is (*L*/*h*_*L*_)^3^, and order of shear force is only (*L*/*h*_*L*_). Hence, the former is far greater than the latter. In this case, the flexural properties can be predicted by stiffness matrix.

The modulus of elasticity is different because cross sections of fin-shaft are not identical. The hydrodynamic force *F*_*E*_ of fin is needed to transform to stress force *F*_*D*_ at *D* of fin-shaft.

Then:

$$F_{D}=\frac{L_{3}+L_{4}}{L_{3}}F_{E}$$ (14)

The stiffness matrix equation of shaft-shell *CD* is established as Eq (13).

$$\left[\begin{array}{c} F_{D}\\ M_{D}\\ F_{C}\\ M_{C} \end{array}\right]=\frac{EI}{L_{2}^{3}}\left[\begin{array}{cccc} 12 & 6L_{2} & -12 & 6L_{2}\\ 6L_{2} & 4L_{2}^{2} & -6L_{2} & 2L_{2}^{2}\\ -12 & -6L_{2} & 12 & -6L_{2}\\ 6L_{2} & 2L_{2}^{2} & -6L_{2} & 4L_{2}^{2} \end{array}\right]\left[\begin{array}{c} v_{D}\\ \phi_{D}\\ v_{D}\\ \phi_{D} \end{array}\right]$$ (15)

Similarly, the stiffness matrix equation of shaft-shell *BC* is established.

$$\left[\begin{array}{c} F_{C}\\ M_{C}\\ F_{B}\\ M_{B} \end{array}\right]=\frac{EI}{L_{3}^{3}}\left[\begin{array}{cccc} 12 & 6L_{3} & -12 & 6L_{3}\\ 6L_{3} & 4L_{3}^{2} & -6L_{3} & 2L_{3}^{2}\\ -12 & -6L_{3} & 12 & -6L_{3}\\ 6L_{3} & 2L_{3}^{2} & -6L_{3} & 4L_{3}^{2} \end{array}\right]\left[\begin{array}{c} v_{C}\\ \phi_{C}\\ v_{B}\\ \phi_{B} \end{array}\right]$$ (16)

The total stiffness matrix of shaft-shell is assembled using direct stiffness method.

$$\left[\begin{array}{c} F_{D}\\ M_{D}\\ F_{C}\\ \begin{array}{l} M_{C}\\ F_{B}\\ M_{B} \end{array} \end{array}\right]=EI\left[\begin{array}{cccccc} \frac{12}{L_{2}^{3}} & \frac{6L_{2}}{L_{2}^{3}} & \frac{-12}{L_{2}^{3}} & \frac{6L_{2}}{L_{2}^{3}} & 0 & 0\\ \frac{6L_{2}}{L_{2}^{3}} & \frac{4L_{2}^{2}}{L_{2}^{3}} & \frac{-6L_{2}}{L_{2}^{3}} & \frac{2L_{2}^{2}}{L_{2}^{3}} & 0 & 0\\ \frac{-12}{L_{2}^{3}} & \frac{-6L_{2}}{L_{2}^{3}} & \frac{12}{L_{2}^{3}}+\frac{12}{L_{3}^{3}} & \frac{-6L_{2}}{L_{2}^{3}}+\frac{6L_{3}}{L_{3}^{3}} & \frac{-12}{L_{3}^{3}} & \frac{6L_{3}}{L_{3}^{3}}\\ \frac{6L_{2}}{L_{2}^{3}} & \frac{2L_{2}^{2}}{L_{2}^{3}} & \frac{-6L_{2}}{L_{2}^{3}}+\frac{6L_{3}}{L_{3}^{3}} & \frac{4L_{2}^{2}}{L_{2}^{3}}+\frac{4L_{3}^{2}}{L_{3}^{3}} & \frac{-6L_{3}}{L_{3}^{3}} & \frac{2L_{3}^{2}}{L_{3}^{3}}\\ 0 & 0 & \frac{-12}{L_{3}^{3}} & \frac{-6L_{3}}{L_{3}^{3}} & \frac{12}{L_{3}^{3}} & \frac{-6L_{3}}{L_{3}^{3}}\\ 0 & 0 & \frac{6L_{3}}{L_{3}^{3}} & \frac{2L_{3}^{2}}{L_{3}^{3}} & \frac{-6L_{3}}{L_{3}^{3}} & \frac{4L_{3}^{2}}{L_{3}^{3}} \end{array}\right]\left[\begin{array}{c} v_{D}\\ \phi_{D}\\ v_{C}\\ \phi_{C}\\ v_{B}\\ \phi_{B} \end{array}\right]$$ (17)

Eq (18) is obtained in conditions of *v*_*C*_ = 0, *v*_*B*_ = 0, *ϕ*_*B*_ = 0:

$$\left[\begin{array}{c} F_{D}\\ M_{D}\\ M_{C} \end{array}\right]=EI\left[\begin{array}{ccc} \frac{12}{L_{2}^{3}} & \frac{6L_{2}}{L_{2}^{3}} & \frac{6L_{2}}{L_{2}^{3}}\\ \frac{6L_{2}}{L_{2}^{3}} & \frac{4L_{2}^{2}}{L_{2}^{3}} & \frac{2L_{2}^{2}}{L_{2}^{3}}\\ \frac{6L_{2}}{L_{2}^{3}} & \frac{2L_{2}^{2}}{L_{2}^{3}} & \frac{4L_{2}^{2}}{L_{2}^{3}}+\frac{4L_{3}^{2}}{L_{3}^{3}} \end{array}\right]\left[\begin{array}{c} v_{D}\\ \phi_{D}\\ \phi_{C} \end{array}\right]$$ (18)

Eq (19) is obtained according to stress force and torque balance *M*_*D*_ = 0, *M*_*C*_ = 0:

$$\left[d]=[\begin{array}{ccc} v_{D} & \phi_{D} & \phi_{C} \end{array}]=[\begin{array}{ccc} -\frac{F_{D}L_{3}^{2}(3L_{2}+4L_{3})}{12EI} & \frac{F_{D}L_{3}^{}(L_{2}+2L_{3})}{4EI} & \frac{F_{D}L_{2}L_{3})}{4EI} \end{array}\right]$$ (19)

Eq (20) is obtained owing to structural characteristics of shaft-core in Fig 3:

$$\phi_{D}=\frac{h_{A}}{L_{1}+L_{2}+L_{3}/2}$$ (20)

The quantitative relationship is established between *h*_*A*_ and *F*_*E*_:

$$h_{A}=\frac{\left(2L_{1}+2L_{2}+L_{3})(L_{2}+2L_{3})(L_{3}+L_{4}\right)}{8EI}F_{E}$$ (21)

Where the annular section inertia *I*_*D*_ at *D* is as shown:

$$I_{D}=\frac{\pi (D^{4}-d^{4})}{64}$$ (22)

Where *D* is outer diameter of shaft-shell. *d* is corresponding inner diameter.

The characteristics of stiffness matrix [*K*] are suitable for finite element generality, which can provide a theoretical basis for other similar problems.

1. [*K*] is a symmetric matrix, which would relate the same number of forces and displacements. Every item is symmetrical, and is in accordance with reciprocation law.
2. [*K*] is a singular matrix. There is no inverse matrix, before applying sufficient boundary conditions to eliminate singularity and prevent movement of rigid body.
3. The terms in main diagonal of stiffness matrix [*K*] are always positive. Otherwise a positive force *F*_*i*_ may induce a negative displacement *u*_*i*_. This case is contradictory to physical characteristic of actual structure.
4. [*K*] is positive semi definite. {*x*}^*T*^[*K*]{*x*} > 0, for nonzero real vector {*x*}.

## 4 Analysis of Measuring Lift

### 4.1 Installation mode of sensors

Installation mode of sensors is designed in order to decompose lift from hydrodynamic force directly. However, the installment and maintenance are extremely difficult in narrow shaft-shell. Hence, a removable shaft-end cover is designed, which is fixed on the end of shaft-shell. The closed space can avoid the entry of sundries and unnecessary interference. Non-contact displacement sensors are installed on the inner side of shaft-end cover, which are difficult to damage. The shaft-end cover can be open while overhaul, which is convenient and practical.

**Fig 5:**
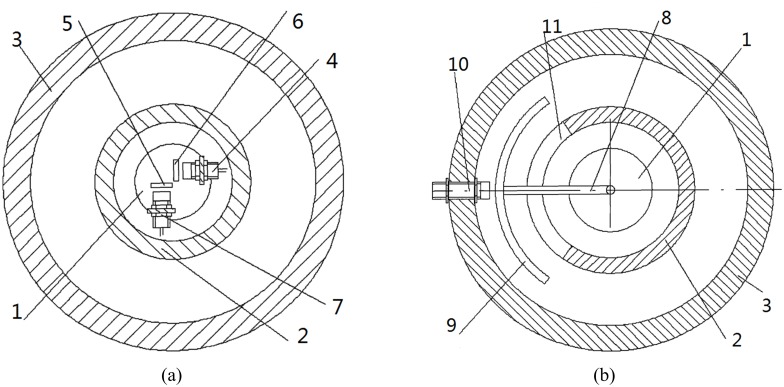
Two installation modes of sensors.

The quantitative relationship is known between lift *L*_*α*_ normal force *N*_*α*_ and tangential force *T*_*α*_ according to Eq (2). Therefore, a sensor is installed in shaft-end cover along normal direction of fin to measure normal displacement of shaft-core. The other sensor is installed along tangential direction to measure tangential displacement. The induction sheets of sensors are installed on the corresponding position of shaft-core as shown in Fig 5(a).

The lifting line is perpendicular to current relative velocity and axis of fin-shaft. Thus single sensor can be improved to measure the displacement of lift direction directly. The sensor is fixed on ship in order to avoid interference of shaft rotation.

The single sensor measurement is designed as above analysis. The transmission rod is installed at the end of shaft-core. A curved cut is arranged on shaft-shell, which can make transmission rod not only pass through shaft-shell, but also do not impact shaft-shell while shaft rotates. The curved induction sheet of sensor is installed on transmission rod. The rotating and bending processes of fin-shaft do not exceed measuring range. Finally, the sensor is fixed on the bearing gland in lift direction as shown in Fig 5(b).

### 4.2 Analysis of main influencing factors

The quantitative relationship between *N*_*α*_, *T*_*α*_ and *h*_*A*_ is obtained using measurement method of double sensors according to Eqs (2) and (21) as shown.

$$h_{A1}=\frac{32(2L_{1}+2L_{2}+L_{3})(L_{2}+2L_{3})(L_{3}+L_{4})}{\pi E(D^{4}-d^{4})}(N\alpha \text{cos}\alpha -T\alpha \text{sin}\alpha )$$ (23)

Similarly, the quantitative relationship between *L*_*α*_ and *h*_*A*_ is obtained using measurement method of single sensor as shown.

$$h_{A2}=\frac{32(2L_{1}+2L_{2}+L_{3})(L_{2}+2L_{3})(L_{3}+L_{4})}{\pi E(D^{4}-d^{4})}L_{\alpha }$$ (24)

Therefore, the factors of affecting shaft-core end displacement are analyzed in order to improve accuracy of measuring lift. There are three factors according to Eqs (23) and (24). First factor is structure length of fin-shaft each part *L*_*i*_. Second factor is material of fin-shaft, which is determined by modulus of elasticity *E*. Third factor is section inertia of fin-shaft *I*_*D*_, which is determined by shape and size of cross section. *I*_*D*_ is determined by outside diameter *D* and inside diameter *d* because section of fin-shaft is circular.

*h*_*A*_ should be as large as possible in order to reduce resolution requirement and selection difficulty of sensors. There are three ways to increase *h*_*A*_ as main factors. The following analysis is carried out with single sensor measurement method as an example.

1. Length of fin-shaft is increased.
  *L*_2_, *L*_3_ and *L*_4_ are related to structural strength. If they are changed, the intensity should be reconsidered. As a result, *L*_2_, *L*_3_ and *L*_4_ can't be easily changed. However, *L*_1_ is the suspended part of shaft-core. *L*_1_ stretches to hull, which does not affect structural strength. *L*_1_ can be properly increased, if the space of cabin is enough.
2. Modulus of elasticity *E* is reduced.
  Modulus of elasticity *E* is determined by material. Material with small modulus of elasticity is selected on the basis of ensuring structural strength.
3. Section inertia *I*_*D*_ is reduced.
  Outer diameter *D* is reduced or inner diameter *d* is increased on the basis of ensuring structural strength. The changes can make smaller section inertia of cross section *I*_*D*_.

### 4.3 Verification and analysis of calculation and simulation

1. Model 1 is set based on design parameters of an actual fin stabilizer installed on ship. The specific parameters are shown in Table 1.
2. *L*_1_ is increased to 1165 *mm* and the other parameters are unchanged on the basis of model 1, which is set to model 2.
3. Alloy structural steel *AISI*5150 of model 1 is replaced with engineering synthetic resins *ABS* in order to compare obviously. Its modulus of elasticity *E* is 2495.9*N*/*mm*^2^ and the other parameters are unchanged, which is set to model 3.
4. Inner diameter is increased to 200*mm*, so section inertia of cross section *I*_*D*_ is decreased. The other parameters are fixed, which is set to model 4.

**Table 1:** Specific parameters of model 1.

NO.	Parameter	Symbol	Numerical value	Units
1	Structure length 1	*L*_1_	165	*mm*
2	Structure length 2	*L*_2_	730	*mm*
3	Structure length 3	*L*_3_	350	*mm*
4	Structure length 4	*L*_4_	1506	*mm*
5	Modulus of elasticity	*E*	204770	*N*/*mm*^2^
6	Outer diameter	*D*	300	*mm*
7	Inner diameter	*D*	140	*mm*
8	Alloy structural steel	*F*	148960	*N*
9	Material of shaft	*AISI*	5150	-
10	Poisson ratio	*λ*	0.29	-
11	Mass density	*MD*	7.8547×10^3^	*Kg*/*mm*^3^
12	Coefficient of thermal expansion	*γ*	1.2816×10^−5^	/°*C*

**Fig 6:**
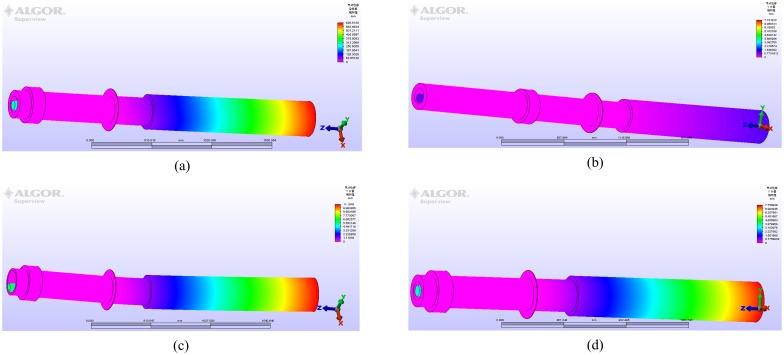
Simulation diagram of each model.

Finite element simulation verification and analysis is carried out. The simulations are shown in Fig 6.

The theoretical calculation is performed based on the given quantitative relation. The results of calculation and simulation can be summarized in Table 2.

**Table 2:** Calculation and simulation results of each model.

NO.	Mold	Variation	Change value	Calculation result	Simulation result	Percentage deviation
1	Model 1	benchmark	165	3.59	3.43	4.45
2	Model 2	structure length 1	1165	6.94	7.06	1.73
3	Model 3	material of shaft	2495.9	294.38	278.06	5.54
4	Model 4	inner diameter	200	6.13	5.91	3.59
5	Units	-	-	mm	mm	%

Following analysis can be obtained by results of comparing calculation and simulation in Table 2:

Deviations of each model are 1.73%, 5.54%, 4.45% and 3.59% respectively by calculated and simulation. They are relatively small, which proves that new device of measuring lift is effective. Moreover, quantitative relation between lift and displacement is correct.

Three main factors of affecting displacement are as follows: structure length of fin-shaft *L*_*i*_, elastic modulus of material *E* and moment of inertia *I*_*D*_. The results are changed while *L*_*i*_, *E* or *I*_*D*_ is changed. Results of calculation and simulation are close, which proves the rationality and accuracy of analysis. Selection difficulty of sensors is reduced due to the large displacement.

There is a small deviation between calculation and simulation, which reasons are as follow. Fin-shaft is assumed to be a slender rigid beam in theory, but actual fin-shaft is complex-shaped and bulky. Meanwhile, moments of inertia are different. The bearings approximate simple supported points. The slope rotation angle and triangle are approximately equal. The multiple sealing position of fin-shaft is stressed. As a result, different interference factors induce deviations actually. The designed device can be calibrated and corrected through quantitative relation and actual mechanical structure in the stage of engineering application. The deviation can be further reduced, which can make device closer to actual project.

## 5 Results and Discussion

### 5.1. Comparison of measuring lift results

#### (1) Experiment of fins in water tank

**Fig 7:**
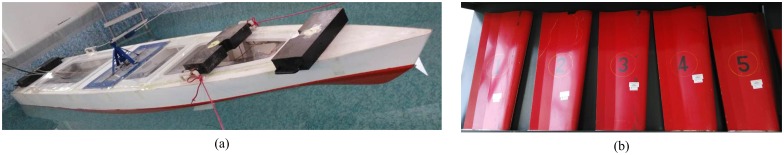
Main experimental equipments in water tank.

As an actual comparison, the dynamic hydrodynamic experiment of fins in water tank is carried out. The main experimental equipments used are shown in Fig 7.

The NACA0015 type fin is chosen to carry out hydrodynamic experiments as an example. The main model parameters of the fin are shown in Table 3.

**Table 3:** Main model parameters of fin.

Parameter	Value	Unit
chord length of root	571.2	*mm*
chord length of tip	316.8	*mm*
fin height	236	*mm*
shaft distances root	254	*mm*
shaft distances tip	182.17	*mm*
sweepback	31.33	*mm*
shaft coordinate	-0.18	*deg*
*l*_*0*_ / 571.2	0.445	*mm*
*l*_*0*_ / 444	0.410	*mm*

**Fig 8:**
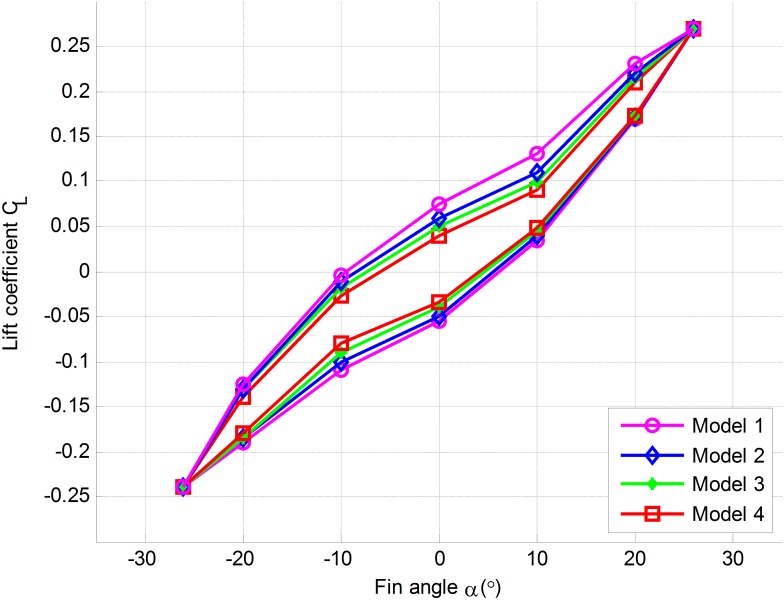
Experimental measuring lift results of fin in water tank.

The experimental results are shown in Fig 8. Here, the model 1 ~ model 4 are the experimental measuring lift results of fin in water tank. The experimental status of fin is shown in Table 4.

**Table 4:** Experimental status of fin.

Mode	Speed	Swing	Swing period	Dimensionless frequency
mode1	3.0	25	2.467	0.06
mode 2	3.0	25	3.700	0.04
mode 3	3.0	25	4.933	0.03
mode 4	3.0	25	7.400	0.02
unit	*m / s*	°	*T*_0_ / *s*	-

#### (2) Measuring lift results of new fin-shaft device

**Fig 9:**
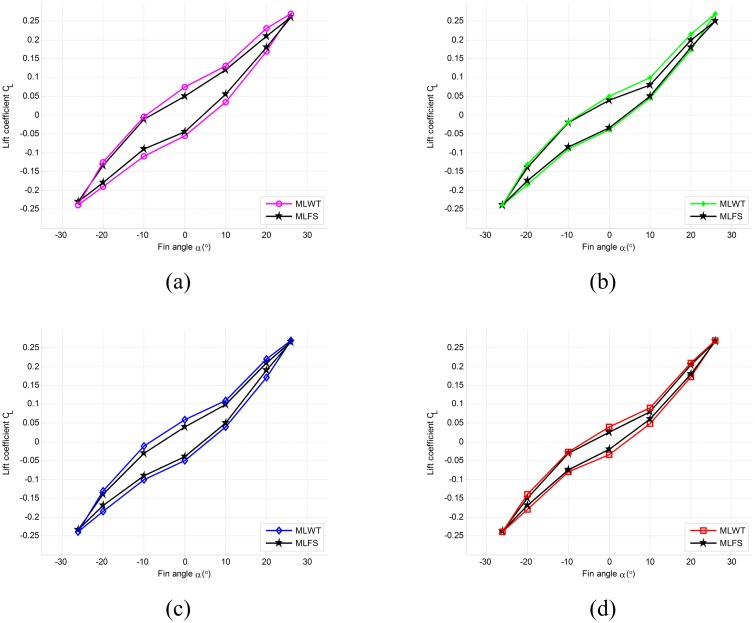
Comparison of measuring lift results.

Under the same conditions, the results are obtained in accordance with the measuring lift method of new fin-shaft provided in this paper. The results of model 1 ~ model 4 are compared with the experimental results in the water tank respectively as shown in Fig 9. Here, MLWT denotes measuring lift results of fin in water tank, and MLFS denotes measuring lift results of new fin-shaft device.

As can be seen from the calculated results of model 1 ~ model 4 in Fig 9, the measuring lift results of new fin-shaft are more accurate than the results of theoretical value calculated by fin angle, which are very close to the actual lift measured in water tank. Therefore, the new measuring lift method can be proved that it is effective. However, the two has a smaller deviation, which is caused by the deviation of transmission and the hypothesis of derivation.

### 5.2 Experimental platform of lift-feedback fin stabilizer

In order to further verify whether this new measuring lift method is applicable to the system of fin stabilizer, the anti-rolling performance is tested by new measuring lift as the system feedback, which is carried out on the experimental platform.

**Fig 10:**
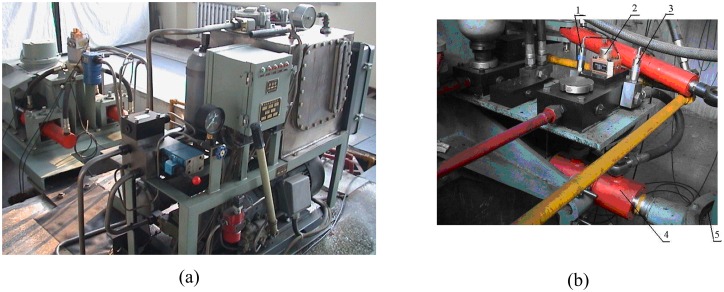
Main experimental device.

The main experimental device used is shown in Fig 10.

#### (1) System structure of fin stabilizer

**Fig 11:**
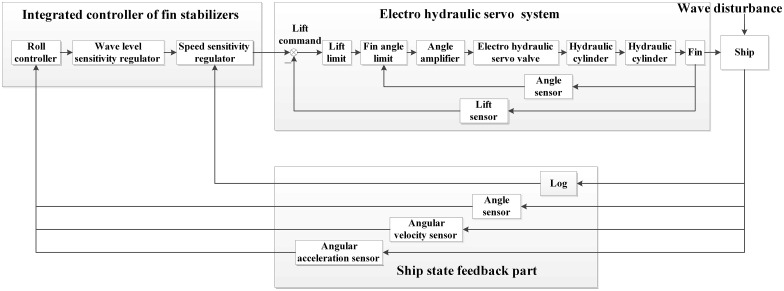
Structure of lift-feedback fin stabilizer.

For the fin stabilizer, the control principle is different between new lift-feedback and conventional fin angle-feedback, so there is a difference in the system structure. The structure of lift-feedback fin stabilizer is three parts: integrated controller, electro hydraulic servo system and ship state feedback part, as shown in Fig 11.

#### (2) Working process

When the ship is subjected to the action of disturbance moment, the sensors of state feedback part can measure roll angle, roll velocity and roll acceleration. The signals are input to data processor, and they are adjusted, calculated and amplified, and then they are transmitted to integrated controller as input quantity.

In the integrated controller, the signal is calculated by control strategy, and the lift needed for fin stabilizer to counter disturbance moment in real time is obtained, which is used as the control signal input to servo system.

The lift control signal is converted to the instruction of turning fin by angle amplifier, which is transmitted to electro-hydraulic servo valve. The hydraulic cylinder drives fin to rotate for the corresponding angle. Due to the effect of fluid dynamics, the control moment is generated to resist disturbance moment by fin. At the same time, the actual lift is measured directly by lift sensor, which is transmitted to servo system controller as a feedback signal.

#### (3) Improved advantage of system feedback

In control form, the lift-feedback fin stabilizer is similar to fin angle-feedback fin stabilizer. But there are essential differences, which are mainly reflected in the following aspects.

1. The output of controller is lift, which is the direct control of ship's control command instead of indirect fin angle command.
2. The effect of speed sensitivity regulator is different. In the fin angle-feedback system, the speed sensitivity regulator is generally placed in the output circuit of controller, and its function is to ensure the stability in the same roll state. And the speed of lift-feedback system is as an input signal of controller. The output of controller is detected by speed sensitivity regulator, which ensures that the output command is always realized. So the system can be guaranteed to work properly.
3. The wave sensitivity regulator can limit the saturation rate of the system to the maximum stable moment, which can avoid the wear of mechanical devices.

#### (4) Comparison of anti-rolling performance

In order to test the performance of the lift-feedback system, a real ship is simulated as an example. Here, the waves are simulated using ITTC single parameter spectrum. The ship parameters are shown in Table 5.

**Table 5:** Main parameters of the ship.

Type	Value	Unit
displacement	1500	*t*
ship length	98	*m*
ship beam	10.2	*m*
draught	3.1	*m*
metacentric height	1.15	*m*
resonant period	7.8	*s*
speed	18	*Kn*
significant wave height	3.8	*m*

Then, the nonlinear roll model with the corresponding lift-feedback fin stabilizer is

$$\ddot{\varphi }=-0.25174\dot{\varphi }-0.7056\left|\dot{\varphi }\right|\dot{\varphi }-0.64836\varphi +15.7696\varphi^{3}-20.65\varphi^{5}-0.00973u-0.39479e^{-7}K_{\omega }$$ (25)

Where *u* is lift control instruction.

In the test, since the practicality of fin stabilizer is considered, the PID controller in actual application is adopted as follow.

$$u_{PID}(s)=(k_{I}\frac{1}{T_{I}s+1}+k_{D}\frac{T_{D1}s}{\left(T_{D1}s+1)(T_{D2}s+1\right)}+k_{p})\varphi (s)$$ (26)

Where, *k*_*p*_, *k*_*I*_ and *k*_*D*_ are the adjustment coefficients of proportion, integral and differential in the controller respectively. In order to solve the integral drift, the integral link is approximated by the inertia link. *T*_*I*_ is time constant. In order to avoid the high frequency disturbance, the differential equation is replaced by the indirect differential link. *T*_*D*1_ and *T*_*D*2_ are the corresponding time constants. Here *k*_*p*_ = 6.90, *k*_*I*_ = 38.7, *k*_*D*_ = 2.06, *T*_*I*_ = 24.607, *T*_*D*1_ = 0.064, *T*_*D*2_ = 0.18.

In order to approach the practical engineering, the simulation is carried out in random waves. Here, the encounter angles are 45°, 90° and 135° respectively, and the speeds of the ship are 9 *Kn*, 18 *Kn*, and 27 *Kn* respectively.

**Fig 12:**
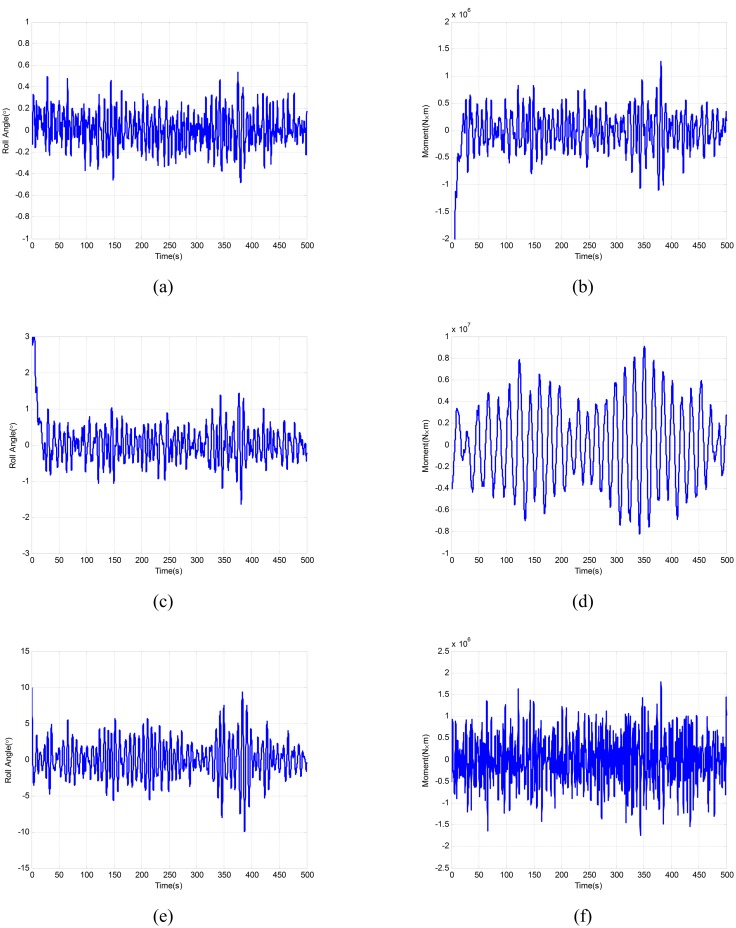
Comparison of anti-rolling performance.

The comparative results of the 18 *Kn* and the encounter angle 45° as an example are as shown in Fig 12. And the statistical results are shown in Table 6.

**Table 6:** Statistical results of anti-rolling performance.

Speeds (Kn)	Encounter angle (°)	Without fin		Fin angle-feedback control			Lift-feedback control		
		mean(°)	variance (°)	mean(°)	variance (°)	roll reduction rate (%)	mean(°)	variance (°)	roll reduction rate (%)
9	45	9.05	4.37	1.94	0.74	78.51	1.67	0.67	81.51
9	90	13.73	6.58	1.69	1.05	87.67	1.57	1.03	88.60
9	135	7.98	3.77	1.08	0.47	86.42	1.00	0.37	87.52
18	45	6.13	2.88	1.06	0.34	82.63	1.03	0.30	83.19
18	90	10.37	4.59	1.11	0.56	89.30	0.99	0.43	90.43
18	135	5.77	2.74	0.70	0.19	87.85	0.61	0.15	89.47
27	45	4.30	1.99	0.53	0.20	87.57	0.51	0.14	88.14
27	90	8.37	3.87	0.83	0.23	90.12	0.67	0.19	92.03
27	135	4.06	1.95	0.41	0.08	89.84	0.39	0.07	90.40

As can be seen from the statistical results, the anti-rolling effect of lift-feedback control is 81.51% ~ 92.03%, and the anti-rolling effect of fin angle-feedback control is 78.51% ~ 90.12%. In different speeds and wave directions, the effect of the former is more effective than the latter. Compared with the traditional fin angle-feedback fin stabilizer, the improved lift-feedback control system can play a better performance. Thus, it is further verified that the measuring lift method proposed in this paper is applicable to the system of fin stabilizer.

## 6 Conclusion

1. Reasons of deviation are analyzed for fin-angle feedback control, which can avoid interferences and rough estimations. The new device of measuring lift is designed on the basis of actual fin stabilizers of ship. It is easy to realize and practical with little changes and inherits the reliability of original equipment.
2. Theoretical analysis of new device is carried out using stiffness matrix based on Euler-Bernoulli beam. Then quantitative relationship between lift and displacement is obtained.
3. A removable shaft-end cover is designed to facilitate installation and calibration of sensors. And closed space can avoid interference from outside. The proposed installation methods of sensors are simple and practical. Relative displacement of shaft-shell and shaft-core is measured by non-contact sensors. Moreover, non-contact sensor is not affected by external force and damaged difficultly. So there are many suitable sensors.
4. The main factors of affecting displacement are analyzed. Then correctness of quantitative relation and influence trend of main factors are proved by calculation and simulation based on design parameters of actual fin stabilizers. The analysis provides theoretical support for engineering design and improvement.
5. Research on lift-feedback control is significant for development of fin stabilizers technology in view of the advantages of innovation, practicality and economy. The application prospects and market value are more potential as a new thing in the field of ship anti-rolling. In addition, there are the extensive applications of rudder, T-hydrofoil, stern board and other many controlled hydrofoils in ship motion control field. To measure accurate dynamic hydrodynamic force directly is a common problem. Thus, the proposed method is a reference for design and improvement of other hydrofoil shafts.
